# Supplementary material for: Study on prediction of blasting cracking radius of liquid CO2 in coal
Source: PLoS One. 2023 Jan 23;18(1):e0280742. doi: 10.1371/journal.pone.0280742 (PMC9870126; doi:10.1371/journal.pone.0280742)
Supplement: S1 Table — (DOCX) [file pone.0280742.s001.docx]

Table. 8 Formula symbol summary table

| Symbols | Implication | Unit |
| --- | --- | --- |
| *ρ* | Density of coal | kg·m^-3^ |
| *u*、*υ*、*w* | Three displacement components of coal mass point | m |
| $\rho\frac{\partial^{2}u}{\partial t^{2}}$, $\rho\frac{\partial^{2}v}{\partial t^{2}}$, and $\rho\frac{\partial^{2}w}{\partial t^{2}}$ | Hree acceleration components of particle | m·s^−2^ |
| *σ_x_*, *σ_y_*, *σ_z_*, *τ_xy_*, *τ_xz_*, and *τ_yz_* | Six stress components of coal (rock) mass point |  |
| *E_g_* | Gas explosion energy | kJ |
| *P*_1_ | Gas pressure in the blasting cracker | 275 MPa |
| *P*_2_ | Standard atmospheric pressure | 0.10108 MPa |
| *V* | Cracking volume | m^3^ |
| *K* | Adiabatic index of the medium |  |
| *Q* | Explosion energy of 1 kg TNT explosive | 4250 kJ·kg^−1^ |
| *x_1_* | Ground stress | MPa |
| *x_2_* | Gas pressure | MPa |
| *x_3_*_,_ | Coal firmness coefficient |  |
| *x_4_* | Gas content | m^3^·t^-1^ |
| *y* | Liquid CO_2_ phase change blasting cracking radius | m |
