# Supplementary material for: Study on prediction of blasting cracking radius of liquid CO2 in coal
Source: PLoS One. 2023 Jan 23;18(1):e0280742. doi: 10.1371/journal.pone.0280742 (PMC9870126; doi:10.1371/journal.pone.0280742)
Supplement: Attachment — Submitted filename: Response to Reviewers.docx [file pone.0280742.s003.docx]

Dear editor:

Thank you very much for giving us an opportunity to revise our manuscript. We appreciate the editor and reviewers very much for their constructive comments and suggestions on our manuscript entitled“**Study on Prediction of Blasting Cracking Radius of Liquid CO_2_ in Coal**”(ID: PONE-D-22-27273).

We have studied reviewers' comments carefully. According to the reviewers' detailed suggestions. we have made a careful revision on the original manuscript. I modified the copy of the markup to form a separate file, named 'Revised Manuscript with Track Changes'.

Kind regards.

Corresponding author: WU Yumo

E-mail address: 13614067811@163.com

Replies to the reviewers’ comments:

Reviewer #2: I hope that the author will make a summary table of all the symbols of the formula, and optimize the curve in the figure, so as to achieve the level of publication.

Response: Because the reviewer pointed out the use of the curve, I corrected and modified all of the curves throughout the manuscript. The visual analysis diagram of the influence of various factors on the fracture radius in the curve of 6 contains four groups of factors: in-situ stress, gas pressure, coal firmness coefficient, and gas content. The units of the four groups of factors on the x-axis are different, but the range of the fracture radius on the y-axis is roughly the same. In order to observe the slope, numerical laws, and other laws, the primary and secondary orders and laws of the factors affecting the fracture radius of liquid CO_2_ phase change blasting are better compared and analyzed. Therefore, the origin software is used to combine the four groups of curves to better reach the effect of visual analysis. According to the standard general drawing format, the following is modified :

Fig. 6 Intuitive analysis of influence of factors on cracking radius

At the request of the reviewer, I created a table that summarized all the symbols in the formula as follows:

Table. 8 Formula symbol summary table

| Symbols | Implication | Unit |
| --- | --- | --- |
| *ρ* | Density of coal | kg·m^-3^ |
| *u*、*υ*、*w* | Three displacement components of coal mass point | m |
| $\rho\frac{\partial^{2}u}{\partial t^{2}}$, $\rho\frac{\partial^{2}v}{\partial t^{2}}$, and $\rho\frac{\partial^{2}w}{\partial t^{2}}$ | Hree acceleration components of particle | m·s^−2^ |
| *σ_x_*, *σ_y_*, *σ_z_*, *τ_xy_*, *τ_xz_*, and *τ_yz_* | Six stress components of coal (rock) mass point |  |
| *E_g_* | Gas explosion energy | kJ |
| *P*_1_ | Gas pressure in the blasting cracker | 275 MPa |
| *P*_2_ | Standard atmospheric pressure | 0.10108 MPa |
| *V* | Cracking volume | m^3^ |
| *K* | Adiabatic index of the medium |  |
| *Q* | Explosion energy of 1 kg TNT explosive | 4250 kJ·kg^−1^ |
| *x_1_* | Ground stress | MPa |
| *x_2_* | Gas pressure | MPa |
| *x_3_*_,_ | Coal firmness coefficient |  |
| *x_4_* | Gas content | m^3^·t^-1^ |
| *y* | Liquid CO_2_ phase change blasting cracking radius | m |
